# INTERGENERATIONAL SOLIDARITY AND MENTAL HEALTH IN CHINESE AMERICAN FAMILIES: A DYADIC APPROACH

**DOI:** 10.1093/geroni/igae098.4282

**Published:** 2024-12-31

**Authors:** Mengting Li, Qun Le, Man Guo, Changmin Peng, Fengyan Tang, Wendi Da, Yanping Jiang

**Affiliations:** Renmin University of China, Beijing, Beijing, China (People’s Republic); University of Massachusetts Lowell, Lowell, Massachusetts, United States; University of Iowa, Iowa City, Iowa, United States; University of Massachusetts Boston, Boston, Massachusetts, United States; University of Pittsburgh, Wexford, Pennsylvania, United States; Rutgers, the State University of New Jersey, New Brunswick, New Jersey, United States; Rutgers, The State University of New Jersey, New Brunswick, New Jersey, United States

## Abstract

Existing family and caregiving studies have focused on mental health of either older adults or adult children, while less is known about the effect of intergenerational relations on mental outcomes for both generations using a dyadic approach. This study examined the association between intergenerational solidarity and mental health among older Chinese Americans and their middle-aged adult children with dyadic analysis, while considering the gendered nature of these relationships. This study included 216 father-child and 337 mother-child dyads. Three indicators of intergenerational solidarity (emotional closeness, contact frequency, and upward support) were assessed by both parents and children. Mental health of both generations was assessed with anxiety, depression, and loneliness. Actor–Partner Interdependence Models were used for dyadic analysis. In father-child dyads, lower contact frequency perceived by children and greater upward support perceived by older fathers were associated with older fathers’ lower levels of anxiety and loneliness; and higher emotional closeness perceived by adult children was associated with adult children’s lower levels of anxiety. In mother-child dyads, higher emotional closeness and upward support perceived by older mothers were associated with older mothers’ lower levels of anxiety, depression, and loneliness, while higher emotional closeness and upward support perceived by adult children were associated with adult children’s lower levels of loneliness. The findings suggest that dyadic intervention promoting emotional closeness and upward support may benefit mental health for both generations and achieve optimal caregiving outcomes. The gendered pattern in intergenerational relations should also be considered in dyadic interventions.

